# Parenting styles, empathy and aggressive behavior in preschool children: an examination of mediating mechanisms

**DOI:** 10.3389/fpsyg.2023.1243623

**Published:** 2023-11-15

**Authors:** Zhumei Lin, Ziqian Zhou, Lijun Zhu, Weige Wu

**Affiliations:** ^1^Department of Psychology, Xiamen Medical College, Xiamen, China; ^2^Second Affiliated Hospital of Xiamen Medical College, Xiamen, China; ^3^Xiamen Xianyue Hospital, Xianyue Hospital Affiliated with Xiamen Medical College, Xiamen, China; ^4^Fujian Psychiatric Center, Fujian Clinical Research Center for Mental Disorders, Xiamen, China

**Keywords:** parenting style, empathy, aggressive behavior, preschool children, authoritarian

## Abstract

**Introduction:**

This study examined the interplay between parenting styles, empathy, and aggressive behavior in Chinese preschool children aged 3–5 years.

**Methods:**

Data were collected from 87 participants using the Child Behavior Checklist, Children’s Empathy Quotient, and Parenting Style Questionnaire, and were subsequently analyzed.

**Results:**

The findings revealed significant age and gender differences in empathy, but not in parenting styles or aggressive behavior. Additionally, a substantial correlation was identified between authoritarian parenting style and aggressive behavior, as well as between children’s empathy levels and aggressive behavior. This indicates that empathy may act as a mediator between parenting style and aggressive behavior.

**Discussion:**

Our findings suggest that an authoritarian parenting style influences aggressive behavior both directly and indirectly through its effect on children’s empathy. These results point toward the possibility that an authoritarian parenting style may stifle the development of empathy in preschool children, subsequently heightening their aggressive behavior.

## 1. Introduction

Aggressive behavior (AB), characterized by an individual’s intentional infliction of physical or mental harm on others or unlawful possession of their property, defies social norms (Perlman and Hirdes, 2010). The majority of studies investigating antisocial behavior prevalence and its progression during early to middle childhood have primarily concentrated on aggressive antisocial behavior. Previous studies have shown that aggression develops at an early age, and that there is a degree of continuity in general aggressive behavior from childhood and adolescence to adulthood (Wahl and Metzner, 2012). An alarmingly high prevalence of aggressive tendencies has been observed in children and has emerged as a pressing public health issue in recent years (Ahemaitijiang et al., 2021). Caims (1979) pointed out that the frequency of aggressive behavior is highest in preschool children and decreases linearly with age. The detection rate of preschool children’s behavioral problems in China reached 14.8% (Yajing et al., 2019), which is higher than the 8–10% reported in Western countries (Wichstrøm et al., 2017). Research on children’s aggressive behavior found that boys were generally more aggressive than girls (Zhang et al., 2003; Yokota, 2017). Aggressive behavior has been identified as an important precursor to numerous social maladjustments, such as juvenile delinquency, peer rejection, and various criminal activities (Reijntjes et al., 2011; Burt et al., 2016).

Parenting style represents the family emotional context in which parent-child interactions take place (Park and Walton-Moss, 2012). The parenting style model developed by Baumrind is the most widely cited. According to a number of relevant dimensions (i.e., acceptance, control, demandingness and disciplinary practices), Baumrind (1968) proposed the three prototypes of parenting style as authoritative, authoritarian and permissive. Authoritarian parents emphasize discipline and obedience. They are less likely to discuss rules with their children and believe in strict adherence to their rules. The parenting environment they provide is distant, cold and limited to one-way communication. In contrast, authoritative parents are more democratic and concerned with explaining the rules and helping the child understand the reasons behind them, rather than strictly following the rules. This is conceptually different from authoritarian parenting in that these parents use two-way communication and reasoning with their children. Based on Maccoby and Martin (1983) proposed a two-dimensional model (parental responsiveness and parental demandingness) and created a typology of four parenting styles: authoritative (parental warmth and parental strictness together), authoritarian (parental strictness but not parental warmth), indulgent (parental warmth but not parental strictness) and neglectful (neither parental warmth nor parental strictness). Based on these models, a considerable number of studies have investigated the influence of parenting on children’s emotional and behavioral development (Perez-Gramaje et al., 2020).

Parenting style has been found to significantly impact the aggressive behavior exhibited by children and adolescents (Lemola et al., 2012; Davies et al., 2019; Qi, 2019). Parental behaviors involving punishment, rejection, or exclusion and other negative parenting styles can induce children and adolescents to mimic delinquent behaviors, intensify hostile emotions and cognitive attributions (Gershoff, 2002; Goulter et al., 2020), stifle and obstruct the growth of emotional and behavioral regulation abilities (Thibodeau et al., 2015; Ganiban et al., 2021), thereby facilitating the emergence of aggressive behaviors (Zhang et al., 2016; Ahemaitijiang et al., 2021). For instance, children who endured severe maltreatment during their early years are prone to display heightened antisocial aggressive behavior (Masud et al., 2019). In contrast, positive parenting styles, such as parental nurturing and appropriate restraint, have demonstrated a deterring effect on antisocial behavior (Criss et al., 2010; Kostulski et al., 2021). Moreover, parenting style not only influences the evolution of children’s social behavior but also their empathy capabilities (Schaffer et al., 2009). Prior researches suggest that parenting serves as a typical extrinsic factor shaping children’s empathy development, with supportive parenting styles exhibiting a positive correlation with children’s empathetic traits (Knafo and Plomin, 2006; Movsesjan et al., 2014; Ma et al., 2020), which are crucial for the progression of aggressive behavior.

Empathy is defined as an individual’s capacity to acknowledge and comprehend the thoughts and emotions of others, and to evaluate and identify problems from others’ perspectives (Decety and Jackson, 2010). It is postulated as a potent mitigator for the development of severe behavioral issues in children (Combe, 2021). Children demonstrating high levels of empathy are more sensitive to others’ emotional states, and this shared emotional recognition can stimulate prosocial behavior while suppressing detrimental actions toward others (Eisenberg et al., 2004; Combe, 2021). Many studies have pointed out that preschoolers’ cognitive and emotional empathy abilities improved significantly with age (Decety and Svetlova, 2012). The main factor influencing the development of emotional empathy in adolescents is the increase in age, and the development of empathy will gradually show gender differences with age. Research on gender differences in empathy has yielded fruitful results. Most studies show that the level of empathy is higher in girls than in boys (Auyeung et al., 2009) while other few studies found no gender differences (Volbrecht et al., 2007). This may be because there are different components of empathy, and the different components may show different gender differences.

While numerous studies have explored the connections between parenting style, empathy, and aggressive behavior, the results have been inconsistent. For example, Miller et al. (2014) found that the impact of various parenting styles on children’s conduct problems depends on their level of empathy. Conversely, Avc and Sak (2021) found no mediating effect of empathy between parenting styles and aggression. Moreover, the majority of these studies primarily focused on adolescents, largely neglecting younger children. Currently, no research has been undertaken specifically on preschool children to examine the intricate relationships between these three factors. Considering that aggressive behaviors in children can surface as early as two years old and rapidly escalate throughout the preschool years (Zhang et al., 2003), the influence of parental behaviors at this stage could present a window of opportunity to modify the trajectory of conduct problems. Therefore, this study aims to investigate the relationships between parenting style, aggressive behavior, and empathy among preschool children. This focus might provide a more effective framework for understanding the mechanisms underlying the development of children’s aggressive behavior.

In this study, we consider a sample of Chinese preschool children aged between 3 and 5 years to explore the effects of parenting style and children’s empathy levels on their aggressive behavior. We also aim to uncover the interaction mechanisms between these influencing factors. Based on a review of the existing literature (Miller et al., 2014), we hypothesize that empathy level acts as a mediator between parenting style and children’s aggressive behavior.

## 2. Materials and methods

### 2.1. Sample and procedure

A total of 94 preschool children aged 3–5 years were selected from a kindergarten in Chengde, China, employing a cluster sampling approach. Parents were given questionnaires, and after excluding incomplete or repetitive responses, we analyzed data from 87 participants, resulting in a response rate of 92.6%. The participant cohort comprised 30 children aged 3 (*M_*age*_* = 3.7, *SD* = 0.19, 53.3% boys), 28 aged 4 (*M_*age*_* = 4.7, *SD* = 0.18, 46.4% boys), and 29 aged 5 (*M_*age*_* = 5.6, *SD* = 0.19, 37.9% boys). The primary caregivers, while at the kindergarten, provided demographic information and completed the questionnaires anonymously. They could withdraw their participation at any stage, and the completed questionnaires were collected immediately. The survey was conducted following obtaining consent from both the kindergarten and the children’s parents or primary caregivers. The Academic Ethics Committee of Xiamen Medical College granted approval for this study.

### 2.2. Measures

#### 2.2.1. Child behavior checklist

The Child Behavior Checklist (CBCL) compiled by Achenbach (2011), was used to evaluate children’s aggressive behavior. This scale, designed for parents, teachers, and children over 10 years old, is a recognized tool for child behavior assessment. The parent form of the CBCL-Aggression Subscale assesses preschool children’s aggressive behavior, featuring 28 items such as “My child physically assaults others.” The scale utilizes a 3-point Likert scale (1 = rarely or never, 3 = most of the time or always), with higher scores indicating higher aggression levels. This study utilized the parent form of the CBCL-Aggression Subscale, demonstrating a high internal consistency (Cronbach’s α = 0.91).

#### 2.2.2. Children’s empathy quotient

The Children’s Empathy Quotient (EQ-C) developed by Auyeung et al. (2009) and translated into Chinese by Peng (2017), was employed to measure children’s empathy. The EQ-C comprises 27 items, e.g., “My child likes to look after other people.” The guardians of the child complete the measure using a 4-point Likert scale (1 = strongly disagree, 4 = strongly agree). Higher scores signify a higher level of children’s empathy. In this study, the Cronbach’s α of the scale was 0.79. Despite poor discrimination for items 9, 15, 22, and 23 [t statistics non-significant level (*p* > 0.05)], these items were not eliminated to maintain comparison consistency with existing literature. The Cronbach’s α of the scale improved (above 0.8) after deleting these four items.

#### 2.2.3. Parenting style questionnaire

Parenting styles were assessed using the Parenting Style Questionnaire compiled by Chen and Luster (1999), encompassing authoritative, authoritarian, and laissez-faire dimensions. However, only the authoritative and authoritarian styles were investigated in this study, reflecting the modern parenting trend where parents prioritize their children’s education and seldom adopt a laissez-faire style (Gong, 2006). The questionnaire includes 40 items such as “I have patience with children.” A 5-point Likert scale (1 = completely non-compliant, 5 = fully compliant) was used. The Cronbach’s α for the authoritative dimension was 0.90, for the authoritarian dimension was 0.85, and the overall Cronbach’s α was 0.80.

### 2.3. Statistical analysis

Data was analyzed using SPSS 20.0. The analysis incorporated multivariate analysis of variance, partial correlation analysis, and multiple regression analysis. Statistical significance was set at *p* < 0.05. Means and standard deviations were used to describe the study variables, and partial correlation coefficients were applied to analyze the correlations between variables.

## 3. Results

### 3.1. Descriptive statistics and variance analysis

Parental educational levels were categorized as follows: 1 = never went to school; 2 = sixth grade or less; 3 = more than sixth grade, but did not graduate high school; 4 = high school graduate; 5 = college or university graduate; 6 = professional training beyond a 4-year college. The most frequent level of education for both fathers and mothers was 4, indicating high school level education. The mean level of average parental education was 4.2 (SD = 0.84), signifying some level of post-high school education. Accounting for parental education as a covariate, the means, standard deviations, and results from the variance analysis are displayed in Table 1. There was a significant effect of age on children’s empathy levels (*p* < 0.01, *?^2^* = 0.21). *Post hoc* tests using Tukey-HSD showed that the empathy levels of 3-year-old children were lower than those of 4- and 5-year-old children (*p_3–4_* < 0.001, *p_3–5_* < 0.01), with no significant difference detected between the 4- and 5-year-old groups. Additionally, the main effect of gender on empathy was significant (*p* < 0.01, Cohen’s *d* = 0.65), suggesting that girls demonstrated higher empathy levels than boys.

**TABLE 1:** Descriptive statistics, variance analysis between variables.

Age	Gender	Parenting style		EQ	AB
		Authoritative	Authoritarian		
3	Boys	14.99 (3.03)	8.96 (2.34)	25.81 (8.30)	13.13 (8.82)
	Girls	15.20 (2.60)	8.81 (2.47)	30.23(5.63)	9.31 (4.79)
4	Boys	16.26 (3.74)	8.64 (1.92)	33.46(8.61)	12.23 (8.08)
	Girls	16.93 (2.55)	7.89 (2.56)	36.93 (4.23)	9.00 (5.32)
5	Boys	16.91 (1.94)	10.18(2.49)	30.00 (5.12)	10.45 (5.7)
	Girls	16.40 (2.03)	8.61 (2.45)	36.35 (5.77)	10.06 (6.85)
	Age	1.81	2.29	7.27**	0.57
*F*	Gender	0.46	3.46	12.20**	2.48
	Age*Gender	0.16	1.93	0.78	0.17

EQ, empathy quotient; AB, aggressive behavior; The same as below. **p < 0.01.

### 3.2. Partial correlation analysis

In the correlation and mediation analyses, gender, age, and parental education were used as control variables. The partial correlation results (see Table 2) indicated a positive correlation between children’s empathy and authoritative parenting style, whereas negative correlations were found with the authoritarian parenting style and aggressive behavior. The correlation between authoritarian parenting style and aggressive behavior was significant, which was not the case for the authoritative parenting style. These results suggest that children’s empathy might mediate the relationship between the authoritarian parenting style (but not the authoritative style) and children’s aggressive behavior.

**TABLE 2:** Correlation between variables.

	Authoritative	Authoritarian	EQ
Authoritative	1		
Authoritarian	−0.28*	1	
EQ	0.48***	−0.42***	1
AB	−0.21	0.42***	−0.33**

*P < 0.05, **p < 0.01, ***p < 0.001.

### 3.3. Mediation analyses

Controlling for gender, age, and parental education, the mediation effect was analyzed using regression (see Table 3). The regression coefficient of aggression on authoritarian parenting style was significant (*c* = 0.43, *t* = 4.07, *p* < 0.001). Additionally, the regression coefficients of empathy on authoritarian style (*a* = −0.40, *t* = −4.10, *p* < 0.001) and aggression on empathy (*b* = −0.36, *t* = −3.10, *p* < 0.01) were significant, suggesting a significant indirect effect. Further analysis showed that the regression coefficient of aggressive behavior on authoritarian parenting style remained significant (*c’* = 0.43, *t* = 4.06, *p* < 0.001), indicating a significant direct effect, with *ab* and *c’* differing in direction. These results suggest that children’s empathy partially mediates the relationship between authoritarian parenting style and aggressive behavior in a negative manner, accounting for 33% of the total effect. Conversely, the regression coefficient of aggression on authoritative parenting style was not significant (*c* = −0.21, *t* = −1.87, *p* > 0.05).

**TABLE 3:** Regression analysis of the mediating effect of empathy.

Predictor variable	Outcome variable	Goodness of fit index		Regression coefficient	
		*R* ^2^	*F*	β	*t*
Authoritarian	AB	0.16	5.59***	0.43	4.07***
Authoritarian	EQ	0.12	9.32***	−0.40	−4.10***
Authoritarian	AB	0.20	5.41***	0.43	4.06***
EQ				−0.36	−3.10**

**p < 0.01, ***p < 0.001.

## 4. Discussion

### 4.1. Child empathy, aggression, and parenting styles: developmental trends

Our preliminary analysis uncovered no significant age or sex differences in parenting styles, mirroring the findings of Gong (2006). Such uniformity could be attributed to contemporary Chinese family structures, typically comprising one or two children, leading parents to invest equally in their child’s upbringing, irrespective of gender. Moreover, since the children in our study were preschoolers, their kindergarten experiences, including learning tasks and other facets, might lack sufficient variability to elicit distinctive parenting styles based on age. Intriguingly, we observed no significant age or gender differences in children’s aggressive behavior either, contrasting with earlier studies (Zhang et al., 2003; Yokota, 2017). We speculate that this may be due to the homogeneity of parenting styles, which fails to delineate differences in aggressive behavior by age or gender.

Contrastingly, significant age and gender differences were observed in children’s empathic tendencies. Specifically, 4- and 5-year-olds exhibited higher empathy levels than 3-year-olds, implying that children’s ability to understand and share others’ emotions improves with age, likely due to maturing cognitive and emotional capacities. Additionally, girls showed higher empathy levels than boys, aligning with prior studies (O’Brien et al., 2013; Sandy et al., 2017; Agnieszka et al., 2020). Such findings imply that gender disparities in empathy emerge as early as ages 3 to 5, potentially stemming from societal expectations of differing gender roles. Cultural norms often encourage girls to be more empathetic and care for others, while boys are urged to be analytical and practical (Lam et al., 2012). According to gender role socialization theory, parents’ rearing pattern of their children is influenced by gender stereotypes, resulting in different parenting needs for boys and girls (Hastings and Derose, 2005). Parents of girls are more likely to emphasize prosocial behaviors and are more likely to talk to girls about other people’s feelings, especially emotional feelings, which will strengthen girls’ ability to be attentive to the needs of others (Coltrane and Adams, 2008). Parents of boys place more emphasis on independence and exploration. As part of the social environment, children are influenced by their parents’ different upbringing, so that both sexes gradually accept different gender role norms in the process of socialization. When women successfully internalize gender roles, they tend to be more sensitive than men to the needs of others in the process of communication, including sensitivity to the emotions of others, especially the negative emotions of others (Jiajin et al., 2010).

The observed gender difference in empathy, despite uniform parenting styles, hints at influences beyond societal role expectations, possibly including inherent physiological factors (Field et al., 2007). Neurobiological research has linked empathy disparities between genders to hormonal factors (Lutchmaya et al., 2002; Honk et al., 2011). For instance, the hormone oxytocin reportedly enhances emotional empathy, while testosterone negatively impacts cognitive empathy, thus contributing to noticeable empathy differences between genders (Chapman et al., 2006; Honk et al., 2011). This biological evidence supports the idea that innate physiological traits partly explain gender differences in empathy. To some extent, the gender differences of empathy in present study may also be attributed to the physiological factors.

### 4.2. Empathy as a mediator

Parenting style is generally considered to have a significant impact on children’s aggressive behavior (Ahemaitijiang et al., 2021). In this study, we found a significant negative correlation between authoritarian parenting style and children’s aggressive behavior, while no correlation was found between authoritative parenting and children’s aggressive behavior. Authoritarian parents who demand absolute obedience from their children, even resorting to threats and punishment, and rarely consider the children’s needs and wishes, may inadvertently breed aggressive behavior. Furthermore, parenting styles are significantly correlated with children’s empathy level, with children’s empathy being positively correlated with authoritative parenting styles and negatively with authoritarian styles. This aligns with existing studies, signifying that different parenting styles yield different impacts on empathy (Jolliffe and Farrington, 2004; Pinquart, 2017).

Authoritative parents get along with children in an accepting and warm way and the encouragement and warmth from parents can promote the development of children’s empathy (Ma et al., 2020). Because in this parenting way, children’s own feelings are accepted and they are more inclined to emotionally understand and accept others. Inversely, when children are treated negatively and rudely, the development of their empathy ability will be negatively affected. Moreover, children’s empathy level also showed a significantly negative correlation with their aggressive behavior, matching previous findings (Shaleh and Binti, 2021; Gómez-Leal et al., 2022). Highly empathetic children are more attuned to others’ emotions and needs, thus promoting prosocial behaviors while avoiding or reducing aggression toward others. On the other hand, children with low empathy are less capable of perceiving emotional information, resulting in more aggressive antisocial behaviors. These results bolster the notion that empathy plays a vital role in fostering moral behavior development and social adaptation in individuals (Vachon et al., 2014).

Previous studies have focused on the influence of parenting style and empathy on children’s aggressive behavior, but this study’s regression analysis results validated that empathy plays a mediating role between parenting style and aggressive behavior in preschool children. Specifically, children’s empathy level plays a partially negative mediating role between authoritarian parenting style and children’s aggressive behavior, consistent with Miller, Johnston, and Pasalich (Miller et al., 2014). The more authoritarian parenting is, the more children’s aggressive behavior will be, and this effect is mediated by the reduction of children’s empathy level due to authoritarian parenting. Overly authoritarian approaches disrupt the intimate balance between children and parents, impacting children’s empathy ability, and thereby increasing children’s aggressive behavior (Yoo et al., 2013).

In current research, there is a controversial subject about which parenting style is associated with better psychosocial adjustment of the child. Contrary to previous suggestions that parental strictness and coercion may be beneficial in raising aggressive adolescents, there are findings that highlight the positive impact of parental warmth without strictness, even with aggressive adolescents (Perez-Gramaje et al., 2020). This may be due to the fact that the subjects were at different ages, which shows that we should adopt different appropriate ways of bringing up children and adolescents. At the preschool stage, parents should minimize authoritarian behavior and emotionally accept children, respect their preferences and opinions, encourage self-expression and respond efficiently to their needs. Nagging parenting styles can provide children with a destructive model for developing empathy, which in turn can increase their aggressive behavior. There’s also research showing that indulgent parenting (warmth without strictness) is associated with equal or better empathy than the authoritative style (warmth and strictness), while non-warm parenting (authoritarian and neglectful) is consistently associated with poor child adjustment (Fuentes et al., 2022). This finding is interesting given the general emphasis in China that parents should not spoil their children. As modern Chinese parents rarely exhibit neglectful parenting styles (Gong, 2006), and indulgent parenting styles were not investigated in this study, future research can explore the relationship between indulgent parenting styles and child behavior in different cultural contexts.

### 4.3. Limitations and future directions

Despite the meaningful findings drawn from this study, there exist some limitations that need to be acknowledged, and these pave the way for future research directions. Firstly, the use of a cross-sectional design hinders the possibility of capturing developmental changes in children’s empathy and aggressive behavior over time or establishing causality between variables. To overcome this limitation, future research could consider adopting a longitudinal design that would provide a dynamic view of these psychological phenomena. And increased collection of demographic information, such as who (mother/father/both) completed the parental survey and the marital status of the parents, could provide additional layers of understanding when interpreting the results.

Secondly, our findings, which are based on data drawn from a single kindergarten’s preschool children, may not necessarily generalize to broader populations. The sample’s homogeneity may limit the universal application of our findings. Future studies should seek to diversify the samples and settings, potentially including multiple kindergartens from different regions or even different countries.

Thirdly, the parent-reported measures used to collect data on parenting styles, children’s empathy, and aggression may be subject to bias and may not accurately represent children’s realities. Future research might employ more direct and diversified data collection methods, such as child interviews, teacher reports, or observational methods, to gain a more comprehensive and accurate understanding of children’s empathy and aggression, as well as parenting styles.

Lastly, the constructs of empathy can be divided into cognitive and affective empathy, which may show different associations with aggression. Accordingly, future research should consider exploring the mediating roles of different types of empathy in the relationship between parenting styles and aggressive behavior. This exploration would deepen the understanding of how specific components of empathy influence children’s aggressive behavior under different parenting styles.

## 5. Conclusion

In conclusion, our research investigated the relationship between parenting style, children’s empathy, and aggressive behavior among Chinese preschoolers aged 3–5 years. The findings demonstrated that an authoritarian parenting style predicts children’s aggression both directly and indirectly, through the mediating role of children’s empathy. We found that as the level of authoritarian parenting increases, children’s empathy development decreases, leading to a rise in aggressive behavior. These findings underscore the critical importance of parenting styles in shaping children’s social-emotional development. They also highlight the pivotal role of empathy as a buffer against aggressive behavior, thereby emphasizing the importance of fostering empathy in early childhood. Our study implies that for healthier child development, it is essential for parents to reduce the rough parenting strategies that restrict empathy in children. This not only promotes social-emotional well-being but can also serve as a powerful tool to minimize aggressive behaviors.

## Data availability statement

The original contributions presented in this study are included in this article/Supplementary material, further inquiries can be directed to the corresponding author.

## Ethics statement

The studies involving humans were approved by the Academic Ethics Committee of Xiamen Medical College. The studies were conducted in accordance with the local legislation and institutional requirements. The participants provided their written informed consent to participate in this study.

## Author contributions

ZL: writing – original draft, investigation, and data curation. ZZ and LZ: data collection and methodology. WW: writing – review and editing. All authors read and approved the final manuscript.
